# Development of a new *Acinetobacter baumannii* pneumonia rabbit model for the preclinical evaluation of future anti-infective strategies

**DOI:** 10.1128/spectrum.01570-24

**Published:** 2024-10-18

**Authors:** S. Albac, N. Anzala, D. Bonnot, C. Djama, P. Chavanet, D. Croisier

**Affiliations:** 1 Vivexia, Dijon, France; 2 Département d’Infectiologie, Centre Hospitalier Universitaire, Dijon, France; Innovations Therapeutiques et Resistances, Toulouse, France

**Keywords:** nosocomial pneumonia, *Acinetobacter baumannii*, pharmacodynamics, resistance, rabbit, innovative model

## Abstract

Carbapenem-resistant *Acinetobacter baumannii* (CRAB) is an emerging cause of hospital-acquired pneumonia (HAP). Preclinical large models are warranted to predict the efficacy and the resistance profile of anti-infectives and mimic how they will be used in the human treatment of CRAB-HAP. Here we reported on the development of an *Acinetobacter baumannii* experimental pneumonia model in immunocompromised rabbits, receiving a 48-h human-simulated regimen. The efficacy of meropenem (2 g/q8h i.v. over prolonged 3-h perfusion), rifampin (25 mg/kg/q8h, i.v.), or the combination of meropenem and rifampin was assessed in rabbits infected with the carbapenem-susceptible *ATCC 17978* reference strain or the CRAB *Turc 2* clinical strain. The emergence of rifampin mutants was also investigated. Meropenem demonstrated a strong pulmonary bacterial reduction in animals infected with the *ATCC 17978* strain (unlike the CRAB strain). The high rifampin dosage was associated with a 1.3 Log_10_ bacterial killing on average but induced the emergence of high-level resistant mutants in 80%–100% of animals, depending on the strain. The adjunction of rifampin to meropenem did not improve the bioburden in the lungs but partially reduced the number of animals exhibiting resistant mutants, whatever the tested strain. However, this adjunctive treatment was insufficient to overcome the emergence of resistance since mutation prevention concentration-related pharmacodynamic indices were unattainable at this dose. This CRAB pneumonia rabbit model represents an innovative tool to evaluate the efficacy of new or existing therapies and will provide informative data on how they can meet the resistance pharmacodynamic targets, which now need to be investigated before deciding on clinical therapeutic regimens.

**IMPORTANCE:**

Within intensive care unit settings, carbapenem-resistant *Acinetobacter baumannii* (CRAB) has emerged as a frequent cause of hospital-acquired pneumonia (HAP) with poor clinical outcomes. This multidrug-resistant pathogen remains very challenging to study in clinical trials, and the U.S. Food and Drug Administration highlighted the limitations of existing small animal models for evaluating antibacterial or prophylactic strategies against such critical infections. These limitations include the difficulty in anticipating the risk of the emergence of resistance during treatment. Here we developed a new *Acinetobacter baumannii* pneumonia rabbit model using high inoculum. We demonstrated the emergence of resistance with rifampin, an existing antibiotic debated as a rescuing option to treat CRAB infections; and even intensified rifampin doses failed to close the mutant selection window. This CRAB pneumonia rabbit model represents a valuable tool to evaluate the efficacy of new or existing therapies and provides supportive data in antimicrobial resistance pharmacodynamics.

## INTRODUCTION

*Acinetobacter baumannii* has become a major health burden in hospital settings and has been ranked at the top of the World Health Organization’s list of critical priority pathogens. Indeed, *Acinetobacter baumannii* has the capability to develop resistance mechanisms to a wide range of broad-spectrum antibiotics, including carbapenems which represent one of the last therapeutic lines active against these infections (1, 2). Carbapenem-resistant *Acinetobacter baumannii* (CRAB) causes various infections (bacteremia, endocarditis, urinary tract infections) but is primarily of concern in hospital-acquired pneumonia (HAP) or ventilator-associated pneumonia (VAP) in critically ill patients (3–6). These infections are associated with significant mortality, from 40% to 70%, and their treatment is challenging because of the current limited therapeutic options (5–7). While awaiting the development and approval of new candidates (8–10), other approaches to optimize therapy, such as the use of existing antibiotic combinations, can also be considered.

In this context, even controversial and far from perfect therapeutic options (11), the beneficial effects of rifampin-based therapies have been repeatedly explored in *in vitro* and mouse models of pneumonia as a last resort in the clinic (7, 12–15). A small number of clinical studies on the synergy between colistin and rifampin or imipenem and rifampin against invasive multidrug-resistant (MDR) *A. baumannii* have also been conducted; however, contradicting microbiological and clinical outcomes have been reported (16–18). The inherent complexity of enrolled patients, the severity of their underlying illnesses, the absence of pharmacokinetic (PK) and pharmacodynamic (PK-PD) investigations, and the use of possibly suboptimal dosing of rifampin are most likely to explain these contradictory outcomes (19).

PK-PD targets for rifampin in the treatment of *A. baumannii* infections have been little explored, partly due to the lack of breakpoints identification from EUCAST or CLSI agencies. However, in 2010, the French Society for Microbiology provided a rifampin breakpoint for *A. baumannii* based on minimum inhibitory concentration (MIC) levels (susceptible if MIC ≤ 4, intermediate if 8 ≤ MIC ≤ 16, and resistant if MIC >16 µg/mL). In 2021, Lee et al. determined the susceptibility of rifampin against a panel of 50 *A*. *baumannii* isolates; the MIC_50_ was 3.13 µg/mL and the MIC_90_ was 6.25 µg/mL (20) .

Based on these values, a few studies published in the literature, including Monte Carlo simulations or clinical studies in critically ill patients, have shown that a standard oral daily dose of 600 mg (equivalent to 10 mg/kg) or even 1,200 mg (equivalent to 20 mg/kg) of rifampin may not be high enough to achieve the presumed therapeutic targets and may contribute to the emergence of resistance (21, 22). These important data raise the question of using higher dosing strategies to improve the probability of success of rifampin in the treatment of MDR *A. baumannii* (Ab) infections, similar to what has been suggested in the treatment of tuberculosis (23). Cohort studies on high doses of rifampin (i.e., 900–2,400 mg/day) confirmed its safety and tolerability, although thoughtful monitoring of any related hepatic adverse effects and other probable complications should be scrupulously carried out (24, 25).

In parallel, the U.S. Food and Drug Administration highlighted limitations of existing small animal models for evaluating antibacterial or prophylactic strategies against critical infections such as HAP caused by *Acinetobacter baumannii* and asked for more diversified and bigger animal models to provide supportive data in the anticipation of clinical outcome (26). In particular, these complementary preclinical models should allow the administration of different dosing strategies (intermittent versus continuous infusion) and the adjustment of the drug exposure to a level similar to that anticipated in humans (often referred to as “humanized dosing”) to better anticipate the PK-PD indices. These models are also expected to recapitulate more accurately the human disease, including similar clinical signs and high bacterial burden, useful to assess the risk of emergence of drug resistance (27, 28).

Here, we developed a neutropenic rabbit model of *A. baumannii* pneumonia, caused by carbapenem-susceptible or -resistant strains and treated with a human equivalent exposure of meropenem alone or in combination with high dosages of rifampin.

Part of these results was presented in Copenhagen at the 33rd European Congress of Clinical Microbiology and Infectious Diseases (ECCMID) 2023 as a paper poster (abstract 05140).

## RESULTS

### MIC and mutant prevention concentration determination

The MIC interpretative criteria determined by EUCAST for meropenem against *A. baumannii* were ≤2 µg/mL (susceptible) and >8 µg/mL (resistant) (29). The *A. baumannii ATCC 17978* strain was susceptible to meropenem (MIC value = 0.25 µg/mL) and the MDR *A. baumannii Turc 2* (OXA-23, *ArmA*) was resistant to meropenem (MIC value >32 µg/mL).

Rifampin MIC values were 8 and 4 µg/mL for the *ATCC 17978* and the *Turc 2* strains, respectively, but EUCAST and CLSI agencies did not establish breakpoints for rifampin against *A. baumannii*. These values were within the range of MIC50/MIC90 values published by Lee et al. (20).

Mutant prevention concentration (MPC) value for rifampin was >1,024 µg/mL against the *ATCC 17978* and the *Turc 2* isolates by plating an inoculum of 10^9^ CFU/plate. The mean mutation frequency of rifampin was 2 × 10^−9^ for these two strains.

### Neutropenia model

**Fig 1:**
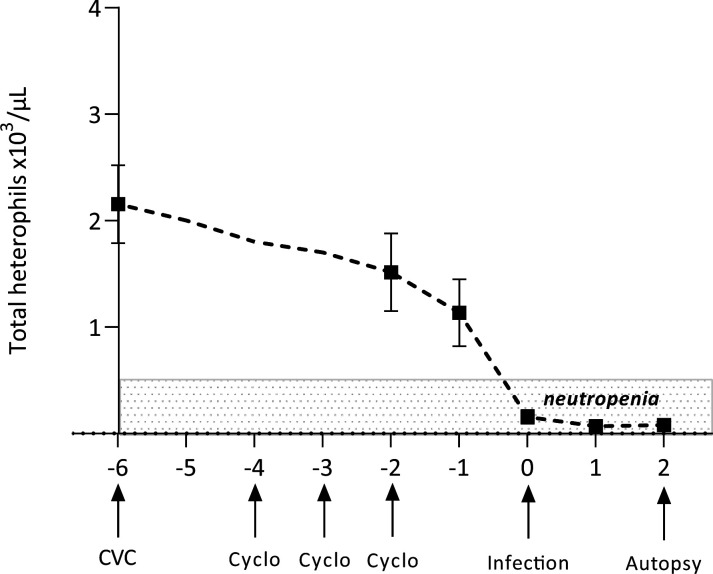
Length and depth of the neutropenia induced in male New Zealand rabbits after three IV doses of 50 mg/kg of cyclophosphamide regimen, expressed as the total number of heterophils (equivalent to neutrophils in humans) in peripheral blood of rabbits (mean ± standard error). CVC = central venous catheter.

As few data were available in the literature on the dose of cyclophosphamide required to engraft bacterial pneumonia in rabbits, different regimens have been assessed. Finally, the neutropenia model in New Zealand rabbits was established using a daily i.v. injection of 50 mg/kg of cyclophosphamide for three consecutive days. The animals generally began to develop symptoms of lack of activity and decreased intake of food and water after the first injection of cyclophosphamide. Dietary supplements were introduced at this stage (hay pallet, i.v. rehydration with physiological serum). Iterative blood samples were collected on day −6,–2, −1, 0, 1, and 2, and leukocytes and heterophiles kinetics were determined (Table 1; Fig. 1). The basal count of leukocytes and heterophiles was determined at day −6, 48 hours before the first dose of cyclophosphamide. On day 0, 2 days after the last injection of cyclophosphamide (corresponding to the day of infection), there was a 93% reduction in heterophils (corresponding to human neutrophils) counts compared to the baseline. Thus, deep neutropenia was achieved at day 0, reached its lowest value at day 1 (0.07 ± 0.05/µL), and remained stable up to day 2 post-infection (time of autopsy).

**TABLE 1:** Total number of leukocytes (cells/mm^3^) and heterophiles in rabbits at baseline (D-6, i.e., 48 h prior to the first dose of cyclophosphamide), during immunosuppression, and at the end of the experiment (D2), compared to reference values for non-immunocompromised rabbits (*n* = 3)

White blood-cell population	Cells 0.10^3^/µL (±SD)						Normal range
	Day −6	Day −2	Day −1	Day 0	Day 1	Day 2	
Total leukocytes	6.19 (1.7)	4.70 (0.4)	3.71 (0.6)	2.07 (0.8)	3.48 (0.3)	4.04 (0.5)	2.9–8.1
Heterophiles	2.15 (1)	1.51 (0.5)	1.13 (0.4)	0.15 (0.1)	0.07 (0.05)	0.08 (0.01)	1.50–3.20

### Pharmacokinetic/pharmacodynamic analysis

#### Pharmacokinetics of meropenem in rabbits

No toxicity was recorded in non-infected rabbits receiving the human-simulated meropenem regimen throughout the observation period.

**Fig 2:**
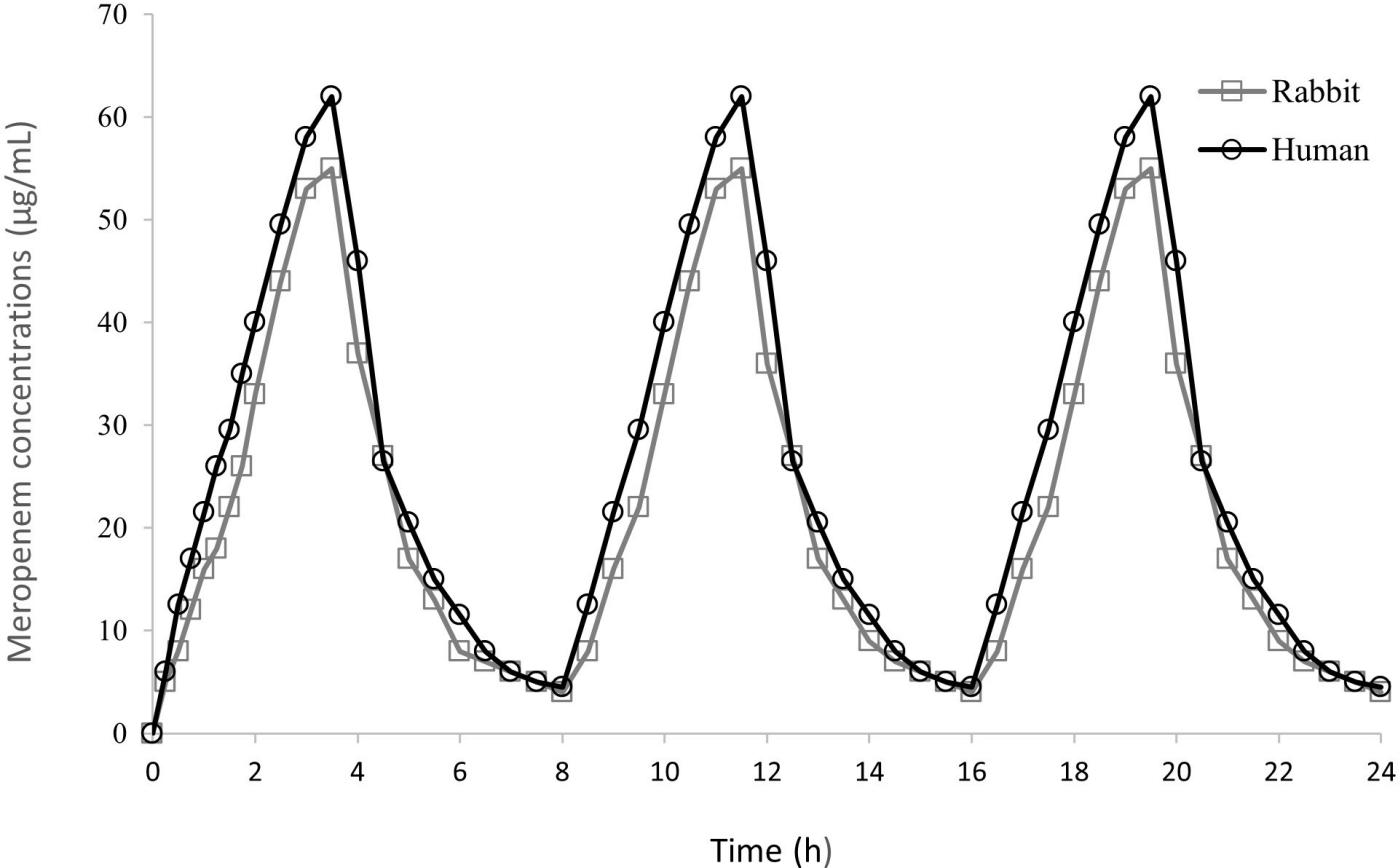
Concentration-time profiles of meropenem (expressed as the total fraction) in the serum of patients receiving a 2 g/q8h by a 3-h infusion (adapted from Jaruratanasirikul *et al.* (30) references) and in uninfected rabbits receiving a humanized dosing regimen.

The raw meropenem PK data expressed as the total fraction are shown in Fig. 2 and in Table 2. The obtained concentration-time curve was generated by integrating all the concentration results, once the human model was obtained. One point on the curve corresponds to the integration of 2 or 3 different samples.

**TABLE 2:** Pharmacokinetic and pharmacodynamic parameters obtained in humans and rabbits following a 3-h infusion of 2 g/8 h of meropenem over a 24-h period^*a*^

PK & PK/PD parameters	Subject	Meropenem 2 g/q8h (3-h infusion)
C_max_ (µg/mL)	Rabbit	65.5
	Human	67 ± 31
C_min_ (µg/mL)	Rabbit	3
	Human	4.3 ± 5
AUC_0-24_ (µg.h/mL)	Rabbit	630
	Human	1,164 ± 660
t_1/2_ (h)	Rabbit	0.8
	Human	1.2 ± 0.4
% T > MIC		
ATCC 17978 (0.25 µg/mL)	Rabbit	100
Turc 2 (>32 µg/mL)		6
ATCC 17978 (0.25 µg/mL)	Human	~100
Turc 2 (>32 µg/mL)		~6

^*a*^ Cmax, maximum serum concentration; Cmin, minimum serum concentration; AUC_0–24_, area under the concentration-time curve from 0 to 24 h; t_1/2_, half-life in serum; %T > MIC, percentage of time that the serum drug concentration was above the MIC.

Overall, the PK parameters obtained in rabbits were similar to those obtained in humans when a human-simulated regimen was applied (30) with an area under the serum concentration-time curve (AUC_0–24_) of 630 µg.h/mL on average and a half-life of elimination of 0.8 h.

The PK/PD parameters for meropenem in the infected model were also calculated to confirm that the predictions were in agreement with the experimental data. The human 3-h infusion of 2 g every 8 h provided serum concentrations above the MIC (%T > MIC) for 100% of the dosing interval when rabbits were infected with the reference strain *ATCC 17978* (meropenem MIC  =  0.25  µg/mL). As expected, the %T > MIC was dramatically reduced (6%) when rabbits were infected with the *Turc 2* strain (meropenem MIC  > 32  µg/mL).

#### Pharmacokinetics of rifampin in rabbits

**Fig 3:**
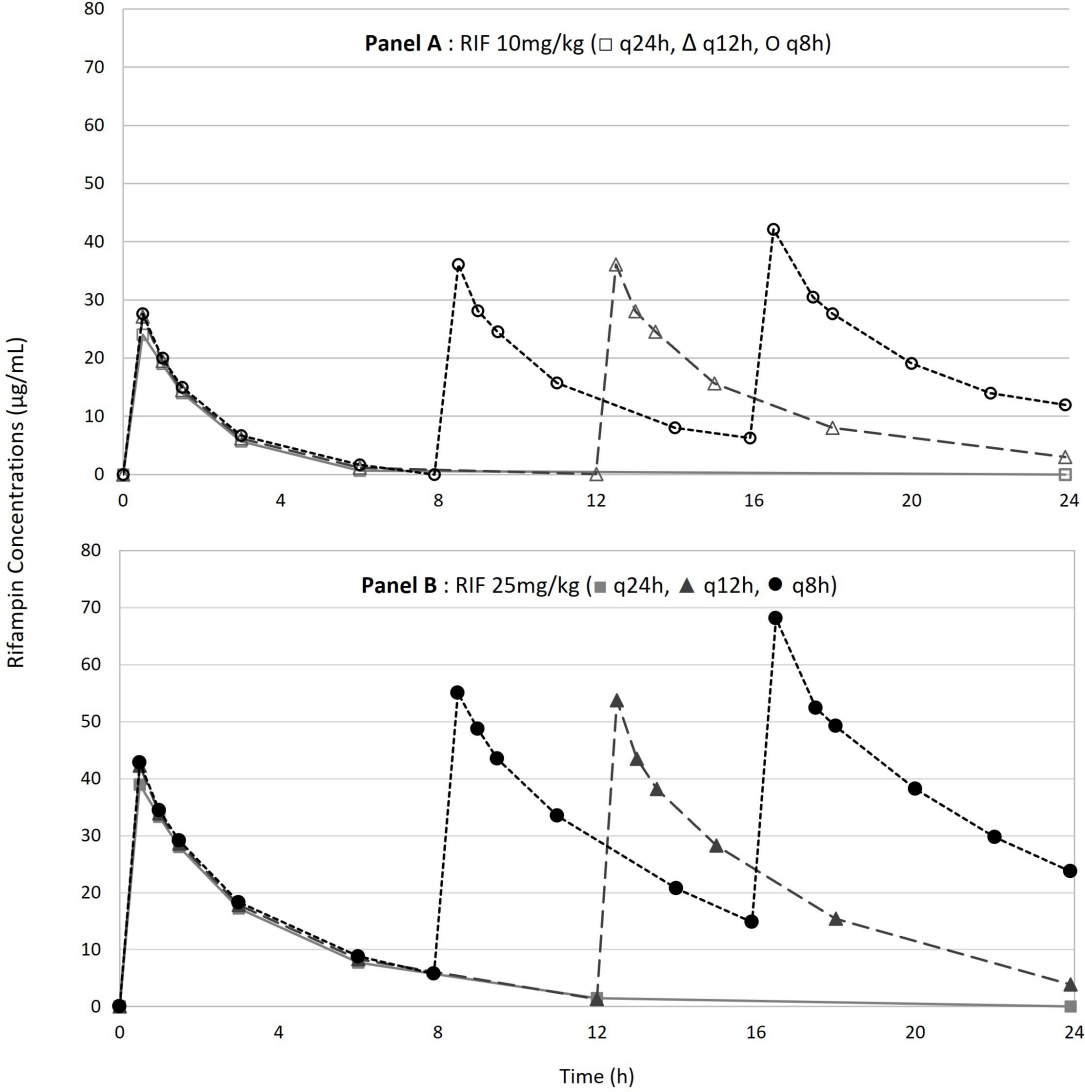
Concentration-time profiles of rifampin (expressed as the total fraction) in the serum of uninfected rabbits receiving a 10 mg/kg or 25 mg/kg rifampin IV injection, administered q24h, q12h, or q8h.

The pharmacokinetics (PK) following a direct i.v. injection over 10 min of 10 mg/kg or 25 mg/kg of rifampin was determined in non-infected rabbits. The animals received either one, two, or three injections, to assess the potential accumulation of rifampin. Animals receiving the first injection of rifampin i.v., whatever the tested dose (10 or 25 mg/kg), presented noteworthy signs that were not listed in the literature (frantic scratching and licking, startles). Surprisingly, these signs disappeared during the following injections, even in the 25 mg/kg/q8h group. The rifampin serum concentration data expressed as the total fraction are shown in Fig. 3 and Table 3. The obtained concentration-time curve was generated by integrating all the concentration results. One point on the curve corresponds to the integration of 2 or three different samples.

**TABLE 3:** Pharmacokinetic and pharmacodynamic parameters obtained in rabbits (compared to existing human data (31)) following an IV bolus (10 min) of rifampin (RIF) at 10 mg/kg or 25 mg/kg, q24h, q12h, or q8h^*a*^

PK & PK/PD parameters	Subject	RIF 10mg/kg/q24h	RIF 10mg/kg/q12h^*b*^	RIF 10mg/kg/q8h^*b*^	RIF 25mg/kg/q24h	RIF 25mg/kg/q12h	RIF 25mg/kg/q8h
**C_max_** (µg/mL)	Rabbit	27.6	36.1	42.1	41.7	53.3	68.1
	Human	5.3 [2.0–23.3]	-	-	14.1 [8.1–29.0]	-	-
**C_min_** (µg/mL)	Rabbit	0	0	0	0	1.7	5.7
	Human	-	-	-	-	-	-
**AUC_0–24_** (µg.h/mL)	Rabbit	54.7	189.5	341.5	144.9	362	694.6
	Human	23.9 [9.1–118.5]	-	-	76.1 [43.5–167.0]	-	-
**t_1/2_**(h)	Rabbit	1	1.5	2.5	1.8	3	4
	Human	1.9 [1.1–4.5]	-	-	2.4 [1.4–3.4]	-	-
C_max_/MIC							
ATCC 17978 (8 µg/mL)	Rabbit	3.4	4.5	5.2	5.2	6.66	8.5
Turc 2 (4 µg/mL)		6.7	9	10.5	10.4	13.3	17
C_max_/MPC							
ATCC 17978 (>1,024 µg/mL)	Rabbit	0	0	0	0	0	0
Turc 2 (>1,024 µg/mL)		0	0	0	0	0	0
AUC/MIC							
ATCC 17978 (8 µg/mL)	Rabbit	7	24	43	18	45	87
Turc 2 (4 µg/mL)		14	47	85	36	91	174
AUC/MPC							
ATCC 17978 (>1,024 µg/mL)	Rabbit	0	0	0	0	0	0
Turc 2 (>1,024 µg/mL)		0	0	0	0	0	0

^*a*^ C_max_, maximum serum concentration; C_min_, minimum serum concentration; AUC_0–24_, area under the concentration-time curve from 0 to 24h; t_1/2_, half-life in serum; %T > MIC, percentage of time that the serum drug concentration was above the MIC; %T > MPC, percentage of time that the serum drug concentration was above the MPC.

^*b*^ “-” indicates not available value.

Overall, the PK parameters obtained in rabbits were in the range of those obtained in patients with tuberculosis and receiving an oral regimen of 600 mg (equivalent to 8–12 mg/kg) or 1,200 mg (equivalent to 17–24mg/kg) (31). Of note, very little or no data are available on the PK of rifampin administered i.v. in humans.

The increased frequency of daily administration of rifampin was accompanied by a significant serum accumulation, in particular when a 25 mg/kg regimen was applied q8h compared to a q24h schedule (the C_min_ increased from 0 to 5.7 µg/mL and the C_max_ increased from 41.7 to 68.1 µg/mL).

Rifampin is a concentration-dependent antimicrobial agent and the PK/PD indices that best predict its antibacterial efficacy are the C_max_/MIC and AUC_0-24_/MIC ratios (21). Jayaram et al. observed that an AUC_0–24_/MIC ≥ 30 µg.h/mL or a C_max_/MIC ≥ 8–10 µg/mL were associated with a 1 Log_10_ CFU/mL bacterial killing in tuberculosis (32).

In light of these data, pharmacodynamic extrapolations were performed in our *Acinetobacter baumannii* pneumonia model (Table 3). The obtained PK/PD simulations (expressed as the total fraction) highlighted that a “standard” regimen using rifampin at a 10 mg/kg dosage, even administered up to q8h, did not reach the C_max_/MIC ≥8–10 target value mentioned above, at least for the *ATCC 17978* strain (MIC = 8 µg/mL). Conversely, increasing the rifampin dosing regimen to 25 mg/kg/q12h or 25 mg/kg/q8h enabled to achieve sufficient exposure for the *Turc 2* strain and the *ATCC 17978* strain, respectively; the 25 mg/kg/q8h was therefore retained for the next treatment phases.

As expected, all PK/PD parameters related to MPC values were dramatically reduced (C_max_/MPC = 0 and AUC_0-24_/MPC = 0) as MPC values were >1,024 µg/mL, whatever the infecting strain.

#### Experimental pneumonia rabbit model

A total of 63 rabbits were used for the whole study. Twenty-seven percent of them died during or just after the immunosuppression procedure, meaning that 46 rabbits were finally included in the analysis: 24 were infected with the *A. baumannii ATCC 17978* susceptible strain and 22 with the *A. baumannii Turc 2* CRAB strain. The number of animals in the control groups was higher compared to the treated groups because usable rabbits from the development phase were included in the analysis and because controls were necessary each time a set of animals was treated.

The mean body weight of infected rabbits decreased throughout the infection period, reaching 16%–20% loss, whatever the control or treated group, and whatever the strain tested (data not shown).

**Fig 4:**
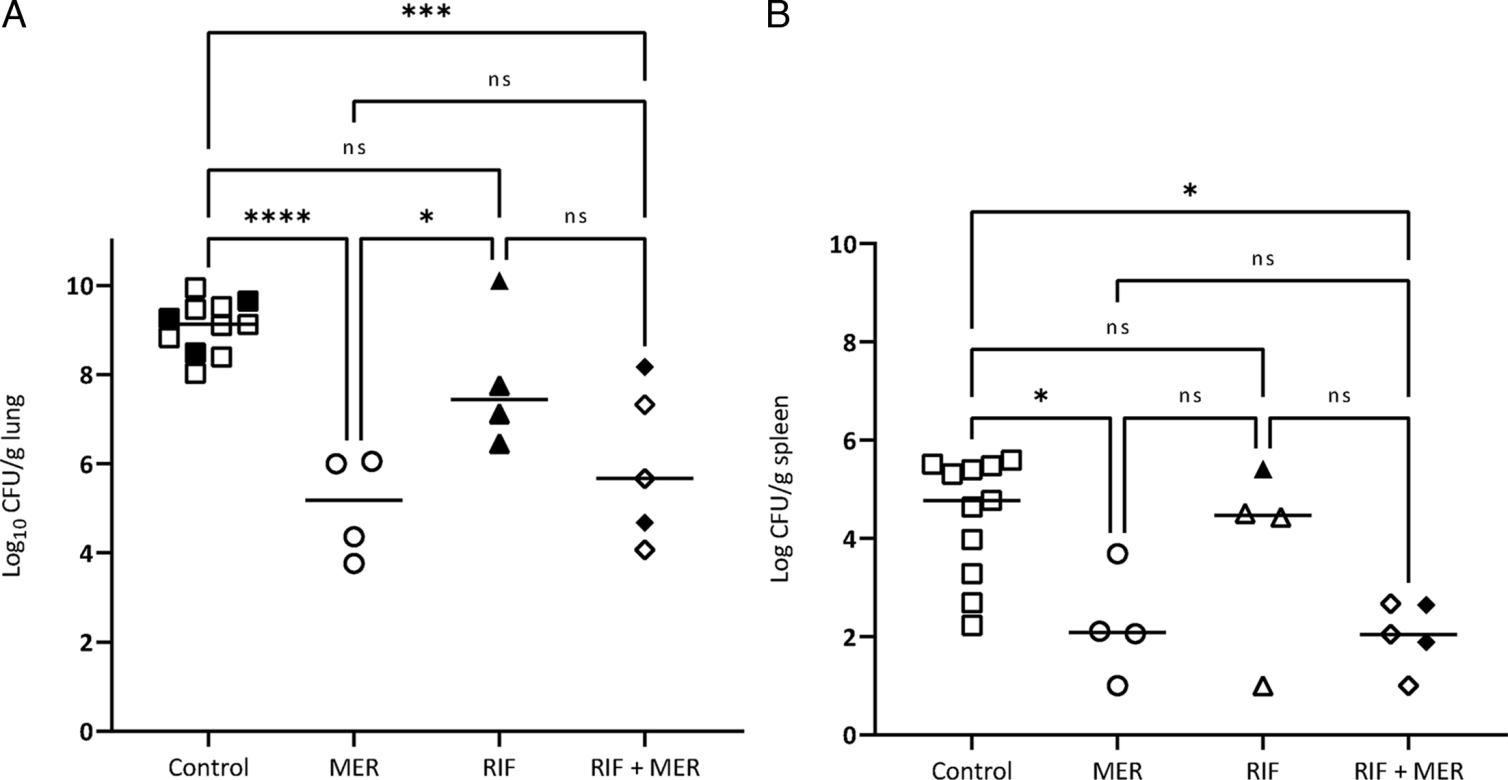
Bacterial burden in lung tissue (**A**) and spleen (**B**) of neutropenic rabbits infected with the *ATCC 17978* strain and receiving a human-simulated treatment with meropenem 2 g/8 h or rifampin 25 mg/kg/8 h for 48 h. Filled symbols correspond to animals carrying resistant mutants and open symbols correspond to animals without resistant mutants. RIF = Rifampin, MER = Meropenem.

**Fig 5:**
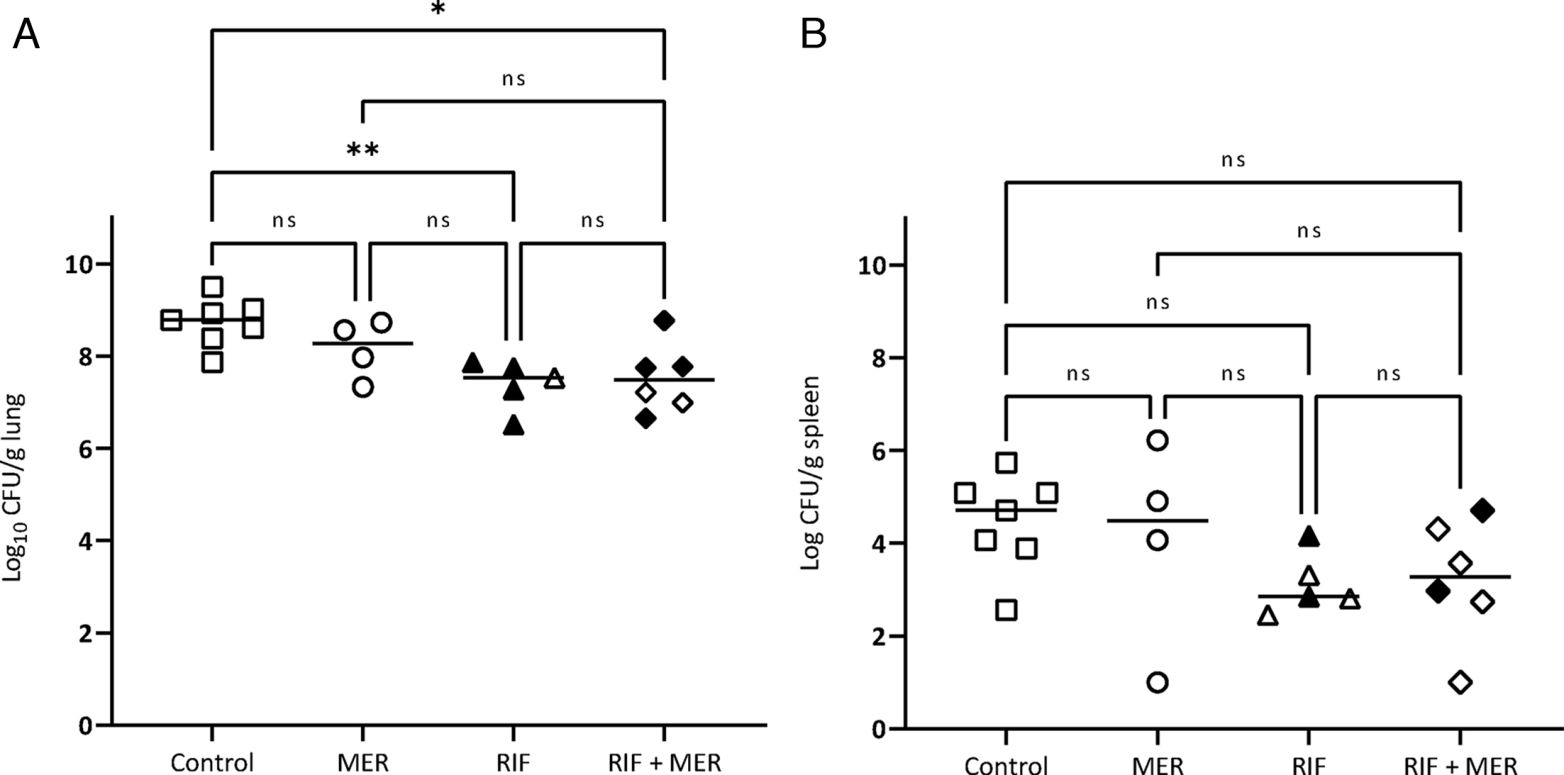
Bacterial burden in lung tissue (**A**) and spleen (**B**) of neutropenic rabbits infected with the *Turc 2* strain and receiving a human-simulated treatment with meropenem 2 g/8 h or rifampin 25 mg/kg/8 h for 48 h. Filled symbols correspond to animals carrying resistant mutants and open symbols correspond to animals without resistant mutants. RIF = Rifampin, MER = Meropenem.

The bacterial concentrations of untreated animals at 48 h post-infection were similar whatever the strain used for the experimental pneumonia, both in lungs (9.07 ± 0.59 Log_10_ CFU/g for the *ATCC 17978* strain and 8.73 ± 0.51 Log_10_ CFU/g for the *Turc 2* strain) and in spleen (4.44 ± 1.2 for *ATCC 17978* and 4.45 ± 1.0 for the *Turc 2* strain) (Fig. 4 and 5).

A significant bacterial reduction was observed in lungs and spleen infected with the *ATCC 17978* strain when animals received a meropenem human-simulated regimen (2 g over a prolonged 3-h period every 8 hours). As anticipated, no antibacterial effect was observed in the lungs or spleen of animals infected with the carbapenem-resistant *Turc 2* strain following a well-conducted meropenem treatment.

In parallel, a significant bacterial reduction in lungs was obtained in animals receiving a 25 mg/kg/q8h rifampin regimen, only in rabbits infected with the CRAB *Turc 2* strain. A downward trend was observed in the lungs of animals infected with the *ATCC 17978* strain, but this was not significant due to the high bacterial load found in one outsider rabbit (despite good permeability of the catheters and therefore good exposure to rifampin). A bacterial reduction was also obtained in the spleen of *Turc 2* infected animals, but this was not significant.

The therapy combining rifampin with meropenem did not demonstrate any additive or synergistic antibacterial effect, whatever the strain tested.

#### *In vivo* mutants

Data related to the rifampin-mutants detection are shown in Fig. 4 and 5 (filled symbols) and Table 4. High-level resistant mutants were recovered from lung homogenates of control animals infected with the *ATCC 17978* strain at a very low level (1.5 Log_10_ CFU/g on average), corresponding roughly to the mutation frequency and confirming that pre-existing rifampin mutants were already present within this high inoculum (~9 Log_10_ CFU/g). No pre-existing rifampin mutants were detected in control animals infected with the CRAB *Turc 2* strain.

**TABLE 4:** Resistant mutants recovered from rabbits infected with the *ATCC 17978* strain or the *Turc 2* strain and receiving the RIF monotherapy or the RIF/MER combination versus control

Strain	Group	Number of rabbits harboring resistant mutants/total of rabbits (%)	[Range] Log_10_ CFU/g
ATCC 17978	Ctrl	3/10 (30%)	[1.56–1.69]
	RIF	4/4 (100%)	[1.12–8.52]
	RIF/MER	2/5 (40%)	[3.66–4.84]
Turc 2	Ctrl	4/7 (57%)	[1.38–1.71]
	RIF	4/5 (80%)	[3.32–6.00]
	RIF/MER	4/6 (66%)	[2.26–8.58]

In total, 80%–100% of rabbits receiving a rifampin monotherapy exhibited resistant mutants when infected by the *Turc 2* or the *ATCC 17978* strain, respectively. The number of resistant colonies, compared to the number of susceptible colonies, varied greatly depending on the animals.

The combined therapy using meropenem and rifampin reduced the number of animals exhibiting highly resistant mutants to rifampin in lungs (40% of positive rabbits, with a mean pulmonary mutants concentration of 4.47 log_10_ CFU/g) compared to the group receiving rifampin as a monotherapy (100% of positive rabbits) when infected by the *ATCC 17978* strain. This percentage was also reduced in *Turc 2* infected animals receiving the combination (66%) compared to the group receiving rifampin alone (80%), but this difference was lower than in animals infected by the *ATCC 17978* strain.

However, the number of mutants was too variable to be able to highlight any significant numerical difference between each group.

## DISCUSSION

*Acinetobacter baumannii* is one of the most predominant pathogens in healthcare-associated infections, and the prevalence of CRAB has notably surged among critically ill patients in recent years. The treatment of HAP and VAP caused by CRAB lacks a unanimous consensus on the optimal antibiotic treatment and poses substantial challenges to clinicians with very limited successful options, leading to a high mortality rate (33). As the clinical development of novel antimicrobial agents progresses slowly, any effort should be also made to optimize the use of already available drugs. However, due to difficulties in enrolling a sufficient number of homogeneous patients suffering from such severe CRAB infections in powered and randomized clinical trials, the major issue remains the current shortage of efficacy data from comparative clinical studies. An observational cohort study including critically ill COVID-19 patients with CRAB-VAP evaluated the efficacy of cefiderocol as monotherapy or as part of antibiotic combination regimens on the 28-day all-cause mortality as the primary endpoint (34). Even if cefiderocol-containing regimens were associated with reduced mortality in CRAB-VAP, the sample size and the observational design limited the study’s conclusions, highlighting the need of future randomized clinical trials (RCTs). Similarly, older attempts have been made to improve the efficacy of molecules such as colistin, by unorthodox antimicrobial combinations using rifampin in treating life-threatening infections due to XDR *A. baumannii*; rifampin-containing regimens did not prove superiority over colistin-monotherapy on the mortality rate (18).

In this context, the development of more predictive animal models than existing small murine models was required by the FDA to optimize the dosing of already licensed and new anti-infective agents and to evaluate their potential to prevent the *in vivo* emergence of resistance, to provide supportive and more secured data upstream of the RCTs (26). Ideally, to mimick the clinical situation observed in pneumonia and examine the development of resistance, these bigger models should enable to reproduce the human PK of tested treatments and to use of high bacterial inoculum. The bacterial quantification is difficult to assess in patients suffering from bacterial pneumonia as it depends on many parameters including the technique used. No “standard” bacterial load is reported but it can range from 10^4^ CFUs/mL to 10^10^ CFUs/mL on average, the highest bacterial loads being closely linked to the severity of lung damage (35).

We thereby developed a novel rabbit infection model of carbapenem-susceptible or -resistant *A. baumannii* pneumonia to assess both the antibacterial efficacy of a human-simulated antibiotic regimen and the emergence of resistance.

A significant mortality rate was recorded in rabbits receiving the immunosuppressive treatment; this early mortality usually occurred in an on-off mode, without warning signs during the close clinical monitoring. The 50 mg/kg/day for 3 days regimen was selected according to the data from the pilot study. A deep and stable neutropenia was not achieved using lower doses of cyclophosphamide; conversely, the administration of higher doses (including those used in mice (36)) was associated with non-humane clinical scores. The literature on the acute toxicity of cyclophosphamide in rabbits is sparse; some authors have reported that high doses of cyclophosphamide considerably increased myocardial toxicity when administered after long-term daunorubicin therapy (37). Other deleterious effects (lipid peroxidation, renal and digestive abnormalities) have been described in a few studies, testing lower and repeated doses of cyclophosphamide (38, 39).

Apart from the mortality induced by immunosuppression, no early mortality was recorded within 48 h post-infection, whether in control or treated animals. The tested strains were previously selected based on their virulence in a murine sepsis model but the gram-negative pneumonia model in rabbits is not suitable for monitoring early mortality. However, the humane endpoints were reached at 48 h post-infection (especially weight loss), suggesting that the tested strains were virulent.

A human-simulated meropenem regimen using a licensed dose of 2 g/8 h over a 3-h period of infusion for 2 days was associated with 100% of T > MIC and a significant bacterial killing (−4 Log_10_ CFU/g on average) in lungs of animals infected by the susceptible *ATCC 17978* strain. Interestingly, this bacterial killing obtained in rabbits, even high, was lower than the bacterial killing observed in our murine model of pneumonia after only 24 h of treatment (50% of T > MIC, −5 Log_10_ CFU/g on average (40)). This may be explained by a lower bacterial load in the lungs of mice (7.5 Log_10_ CFU/g on average) compared to rabbits (9 Log_10_ CFU/g on average) at the initiation of treatment. The pulmonary microscopic damage may also be different between the two models and may be associated with different penetration rates of meropenem within the epithelial lining fluid. As expected, a meropenem human-simulated regimen had no antibacterial effect in the lungs of rabbits infected by the CRAB strain. Altogether, these data validate the *A. baumannii* pneumonia model in rabbits and also suggest that this rabbit model provides data that may differ from that obtained in a mouse model, at least in terms of bacterial killing.

However, the CRAB pneumonia model in rabbits had to be challenged with a positive control to be fully validated. Similarly to difficulties encountered by clinicians in combatting these superinfections, we faced extremely limited therapeutic (open access) options at the time of this preclinical study. The antibiogram obtained for the CRAB strain showed that only colistin remains active *in vitro* (MIC = 0.5 µg/mL). However, except for nebulized, the efficacy of intravenous colistin in the treatment of HAP caused by multidrug-resistant gram-negative bacteria has been debated because of its poor penetration into the pulmonary parenchyma (41). Therefore, colistin was not retained as a positive control in this rabbit model. Although unconventional and debated, rifampin had been discussed as a last resort in the clinics (due to both its bactericidal properties and its capacity to induce the emergence of resistance) and was selected to be further investigated, alone or in combination with meropenem, in the treatment of this CRAB pneumonia model.

By administering high doses of rifampin (25 mg/kg/q8h) to achieve the expected exposure defined in our MIC-based PK/PD pilot study, a 1.2 to 1.4 Log_10_ CFU/g bacterial killing was recorded in the lungs of rabbits, depending on the strain. But this “boosted” regimen failed to meet therapeutic success, due to the emergence of highly resistant mutants in a large proportion of animals. These observations are in line with the MPC values obtained *in vitro* (>1024 µg/mL), which suggest that the PK/PD targets for prevention of resistance (or extinction of pre-existing mutants) are clinically unachievable, even at these high doses (and which could no longer be increased further for tolerance reasons). Similarly, *in vitro* MPCs of rifampin for *E. coli* (>4,000 µg/mL) and colistin for *A. baumannii* (64 µg/mL) are so high that they cannot be exceeded *in vivo* (42, 43). Also, the high bacterial densities, characteristic of this *A. baumannii* rabbit model, can be associated with pre-existing resistant mutants in control animals, meaning that the resistant mutants are likely to be enriched by antibiotic treatment.

In comparison, results from a previous *in vivo* work obtained in a *parC*-mutant *S. pneumoniae* pneumonia rabbit model indicated that the administration of moxifloxacin at two times the licensed dose enabled the extinction of high-level fluoroquinolone resistance. In this case, a higher (but acceptable) moxifloxacin regimen was able to close the MSW, given that the number of mutants recovered dropped sharply as the time the serum moxifloxacin concentration exceeded the MPC (the upper boundary of the selection window (28)). Similar observations were reported more recently in a meropenem-susceptible wild-type *Pseudomonas aeruginosa* rabbit model of nosocomial pneumonia, in which the intensification of a meropenem regimen or the co-administration of meropenem/amikacin reduced the rate of resistance (27).

Interestingly, our internal data obtained in the CRAB pneumonia murine model receiving a 25 mg/kg/q8h rifampin regimen for 24 h (and even lower doses) demonstrated a huge fall in bacterial load, without any resistant mutant (internal data). Again, these findings highlight the major discrepancies that can exist between different preclinical models (bacterial load at the initiation of the treatment, penetration of the antibiotic at the site of infection, use of mucin or not, route of bacterial inoculation, type of lung damage, etc.). This offers evidence for the importance that should be attached to investigate each of them, from a clinical development perspective.

The objective of this study was also to evaluate whether the combination of meropenem and rifampin could reduce or close the gap of mutant selection window (MSW) of individual antibiotics against carbapenem-susceptible or -resistant *A. baumannii* in this pneumonia rabbit model. The overall results indicate that the combination reduced by 60% the number of animals exhibiting highly resistant mutants to rifampin in the lungs when infected with the carbapenem-susceptible strain. However, there was no additional or synergistic antibacterial effect of the combination and 40% of rabbits still developed resistance. Even more notably, the therapeutic combination only slightly reduced the proportion of rabbits carrying highly resistant mutants to rifampin.

Our study has also limitations worth considering. First, due to a very limited number of available and active therapies against this type of super-infection, we only tested rifampin as a last resort treatment. Even a high-dose rifampin regimen was inappropriate in the treatment of susceptible or multiresistant *A. baumannii*-induced pneumonia as it could not mitigate treatment failure, this study provides a strong framework for pharmacodynamic evaluation of drug-resistant mutant emergence and supports the mutant selection-window hypothesis, an idea that provides a rationale for restricting mutant growth. Second, a limited number of animals was included in each treatment arm, but the group size was sufficient for a reliable statistical analysis. Regardless, with enough information, using more animals was not ethically justified, considering the severity of the model. Third, some pharmacokinetic investigation should have been performed in infected animals considering that the PK profile of anti-infectives can be modified due to a severe infection, as is the case for HAP. In addition, the free fraction has not been assessed in the PK/PD analysis. According to the literature, meropenem plasma protein-binding ratios in rabbits and humans were similar (even lower in rabbits, 12% on average, depending on plasma concentrations) (44). If this protein-binding ratio is applied to our study, PK/PD values remain similar (100% *f*T >MIC for the ATCC strain and 4% *f*T >MIC for the CRAB strain). Rifampin displays higher protein binding than meropenem (72%–91% for humans and 80%–85% for rabbits) (45). However, this ratio can significantly vary according to plasma concentrations, suggesting that the free fraction should have been assessed for each sample individually. Finally, this model is a non-ventilated model, that probably better to mimic the human pathology than murine models but that does not reproduce the entire pathogenesis of the disease during mechanical ventilation. The development of a CRAB VAP model will likely be needed but will require even more infrastructure and funds than for the non-ventilated pneumonia model.

Despite these limitations and beyond the rifampin story which rather served as a benchmark here, this *A. baumannii* pneumonia model in neutropenic rabbits represents a valuable and innovative tool that should be now considered by drug developers for the assessment of antimicrobial agents. In the era of the fight against anti-microbial resistance, this model, which studies the disease in its most severe form and is relevant to the human disease manifestation, will be useful to establish old/new antibacterials dosing to successfully treat CRAB pneumonia and prevent the emergence of resistance, thus derisking the randomized controlled trials for the treatment of *A. baumannii* pneumonia in critically ill patients.

## MATERIALS AND METHODS

### Bacterial strains

Two *A. baumannii* isolates were used in these experiments: the *A. baumannii* ATCC 17978 reference strain and the clinical MDR *A. baumannii* Turc 2 isolate, kindly provided by P. Nordmann & L. Poirel (University of Friburg, Switzerland), originally recovered from a blood culture of a patient suffering from pneumonia and producing an oxacillinase (OXA-23) and a methylase (*ArmA*). Bacterial stocks were kept at −80°C in cryobeads (bioMérieux, Marcy l’Etoile, France).

### Antimicrobial agents

For *in vitro* experiments, meropenem and rifampin were purchased from Sigma Aldrich (Saint Quentin Fallavier, France). For *in vivo* studies, injectable commercial formulations were reconstituted according to the manufacturer’s instructions: Meropenem (1 g, PanPharma) and Rifadine (600 mg Sanofi-Aventis).

### MIC and MPC determination

The MICs of all antimicrobial agents were tested by twofold serial dilutions using the broth microdilution method according to EUCAST recommendations. The inoculum suspension was adjusted in sterile water to a 0.5 McFarland suspension and subsequently diluted in cation-adjusted Mueller-Hinton Broth so that each well contained approximately 5 × 10^5^ CFU/mL. Antibiotic solutions were prepared following a proper dilution in Mueller Hinton Broth of stock solutions stored at −80°C after reconstitution following the manufacturer’s instructions. All microdilution plates were incubated at 37°C for 18 h in an ambient air incubator right after adding the inoculum, adjusting the final volume to 100 µL per well, and careful sealing with a plastic film to prevent drying. Each MIC experiment was performed in triplicate.

*In vitro* rifampin MPC (defined as the concentration required to block the growth of all single-step-mutant subpopulations) determination was performed according to the protocol described by Zhao and Drlica (42). MH plates containing rifampin at various concentrations (including the MIC value as the lowest concentration to 1,024 µg/mL as the highest concentration) were inoculated with 100 µL of a 10^10^ CFU/mL bacterial suspension (10^9^ CFU/mL per plate). MPC was recorded as the lowest antibiotic concentration that prevented bacterial colony formation after 72 h of incubation at 37°C in ambient air. In addition, an enumeration of mutant colonies was performed to assess the mutation frequency.

### Animals and ethical aspects

Male New Zealand White rabbits (body weight, 2.9–3.6 kg) were obtained from Elevage Scientific of Burgundy university (Dijon, France). These animals were placed in individual cages located in a level 2 microbiological area and were nourished *ad libitum* with drinkable water and feed, according to current recommendations. The housing environment was enriched with toys and hay bales to avoid animal boredom. The experimental protocol was approved by the local ethics committee for animal experimentation of Grand Campus Dijon, Bourgogne, France, and the French Minister of Research and Teaching (APAFIS#37831-2022062914061920 v1) and performed in accordance with the current recommendations of the European Institute of Health EU Directive 86/609. All efforts were made to minimize the suffering of animals.

### Experimental neutropenic pneumonia in rabbit model

A two-lumen central venous catheter (CVC) was surgically placed into the jugular vein under deep anesthesia (ketamine 60 mg/kg and xylazine 5 mg/kg, intramuscular route) and analgesia (buprenorphine 0.05 mg/kg, subcutaneous route) through a lateral incision of the neck, then a subcutaneous tunneling through the interscapular area (46). The first lumen was used to subsequently infuse cyclophosphamide and antibiotics, the second one was allocated to blood sampling. Two days after the surgical procedure, animals were immunosuppressed following a daily i.v. injection of cyclophosphamide (Baxter, 1 g, France) at a dose of 50 mg/kg for three consecutive days. The rabbit’s general conditions including eating, drinking, activity, behavior, diarrhea, and mortality were daily monitored. Total leukocytes and heterophiles (similar to neutrophiles in the human body) were monitored by Cerbavet (Massy, France) in blood samples. Forty-eight hours after the last injection of cyclophosphamide, the bacterial pneumonia was induced under deep anesthesia (ketamine 60 mg/kg and xylazine 5 mg/kg, intramuscular route) by the endobronchial challenge of the animals with 1 mL of saline containing 50% vol/vol of mucin (10%) and 10^10^ CFU/mL of either tested strain. Five hours after endobronchial inoculation, rabbits were randomly assigned to controls (saline serum) or to a human simulated exposure of one of the tested antibiotic treatments for up to 2 days: meropenem 2 g/q8h/48-h to 3-h infusion I.V or rifampin 25 mg/kg/q8h/48 h I.V or the combination meropenem + rifampin.

### Humanized meropenem dosing regimen

To mimic the concentration-time profile of meropenem in patients receiving 2 g every 8 h as an extended 3 h infusion (30), three catheterized rabbits were treated with meropenem using a staggered-continuous infusion protocol driven by programmed syringe pumps. The calibration of the different sequences was carried out in uninfected animals. A single animal could receive different meropenem sequences (four maximum) separated by a wash-out period until the desired human exposure was reached. Serial blood samples (maximum 0.5 mL per sample) were collected from the CVC at several timepoints, within the limits of maximal blood volumes authorized by the ethical rules. The antibiotic concentrations in serum were determined in triplicate on iterative blood samples using a disc plate bioassay with *Escherichia coli NIJJHC2* as the indicator organism. The limit of detection was 0.2 µg/mL. Standard curves were established with solutions (progression from 0.5 µg/mL to 10 µg/mL) in serum. The linearity of the standard curves used for disc plate bioassays was at least 0,97 (*r*^2^) and the mean coefficient of variation was 7%.

### Rifampin dosing regimen

The PK following a direct i.v. injection over 10 min of 10 mg/kg or 25 mg/kg of rifampin was determined in non-infected rabbits. The animals received one, two, or three injections of either 10 or 25 mg/kg, to assess the potential accumulation of rifampin. With a view to reducing the number of animals used, a single animal could receive different rifampin regimens separated by a wash-out period. Serial blood samples (maximum 0.5 mL per sample) were collected from the CVC at several timepoints (6 to 8 samples per rabbit), within the limits of maximal blood volumes authorized by the ethical rules. Bio-analysis was performed using an HPLC/MS-MS method, according to the ISO 15189 standard (Laboratoire de Pharmacologie, CHU Amiens, France). *The chromatographic conditions were as follows: Instrument Nexera Shimadzu, Column Kinetex biphenyl, 150 × 4,6 mn, particle diameter 5 µm, run time 12.5 min, linearity of the method between 0 and 50 µg/mL, quantification wavelength 330 nm*.

### Pharmacokinetic/pharmacodynamic analysis

For each tested antibiotic, the sampling strategy enabled estimation of peak concentration (C_max_), total exposure (area under the serum concentration versus time up to 24 h, AUC_0–24_), and half-life of elimination (t_1/2_). The following PK/PD parameters related to MIC were calculated: C_max_/MIC ratio; AUC_0–24_ > MIC ratio; and percentage of time above the MIC (%T > MIC).

### Evaluation of infection

Rabbits were anesthetized (ketamine 30 mg/kg and xylazine 2 mg/kg, iv) and sacrificed by an overdose of pentobarbital (135 mg/kg, iv) after a 2-day period of treatment and 5 h after the end of antibiotic infusion to avoid any carry-over effect. The spleen and each lung (right and left) were weighed and homogenized in sterile water. Bacteria were counted in a sample of this crude homogenate by plating 10-fold dilutions on Drigalski agar plates (bioMérieux, France) and incubating the plates for 24 h at 37°C; the detection threshold was 1 Log_10_ CFU/g. Bacterial concentrations in each lung and the spleen were determined after adjusting for weight. For each rabbit, the mean pulmonary bacterial concentration was calculated using the bacterial concentration of each lung (expressed as Log_10_ CFU/g).

The emergence of less rifampin-susceptible *A. baumannii* colonies was systematically investigated by plating lung and spleen homogenates on media containing rifampin at a concentration equivalent to 3 × MIC.

### Statistical analysis

All data are expressed as mean ± standard deviation. For statistical comparisons of the differences between residual bacterial densities, culture-negative samples were considered to contain 1 Log_10_ CFU/g. The statistical significance of differences between groups was determined by the one-way ANOVA followed by a *post hoc* Bonferroni test using Graphpad Prism 7.0 (Graph Pad Software, San Diego, CA).

## References

[1] Maragakis LL, Perl TM. 2008. Acinetobacter baumannii: epidemiology, antimicrobial resistance, and treatment options. Clin Infect Dis 46:1254–1263. doi:10.1086/529198 18444865

[2] Poirel L, Bonnin RA, Nordmann P. 2011. Genetic basis of antibiotic resistance in pathogenic Acinetobacter species. IUBMB Life 63:1061–1067. doi:10.1002/iub.532 21990280

[3] Cisneros JM, Rodríguez-Baño J. 2002. Nosocomial bacteremia due to Acinetobacter baumannii: epidemiology, clinical features and treatment. Clin Microbiol Infect 8:687–693. doi:10.1046/j.1469-0691.2002.00487.x 12445005

[4] Peleg AY, Seifert H, Paterson DL. 2008*.* Acinetobacter baumannii: emergence of a successful pathogen. Clin Microbiol Rev 21:538–582. doi:10.1128/CMR.00058-07 18625687 PMC2493088

[5] Garnacho-Montero J, Ortiz-Leyba C, Fernández-Hinojosa E, Aldabó-Pallás T, Cayuela A, Marquez-Vácaro JA, Garcia-Curiel A, Jiménez-Jiménez FJ. 2005. Acinetobacter baumannii ventilator-associated pneumonia: epidemiological and clinical findings. Intensive Care Med 31:649–655. doi:10.1007/s00134-005-2598-0 15785929

[6] Ibrahim S, Al-Saryi N, Al-Kadmy IMS, Aziz SN. 2021. Multidrug-resistant Acinetobacter baumannii as an emerging concern in hospitals. Mol Biol Rep 48:6987–6998. doi:10.1007/s11033-021-06690-6 34460060 PMC8403534

[7] Montero A, Ariza J, Corbella X, Doménech A, Cabellos C, Ayats J, Tubau F, Borraz C, Gudiol F. 2004. Antibiotic combinations for serious infections caused by carbapenem-resistant Acinetobacter baumannii in a mouse pneumonia model. J Antimicrob Chemother 54:1085–1091. doi:10.1093/jac/dkh485 15546972

[8] Karruli A, Migliaccio A, Pournaras S, Durante-Mangoni E, Zarrilli R. 2023. Cefiderocol and Sulbactam-Durlobactam against Carbapenem-Resistant Acinetobacter baumannii. Antibiotics (Basel) 12:1729. doi:10.3390/antibiotics12121729 38136764 PMC10740486

[9] Trebosc V, Gartenmann S, Tötzl M, Lucchini V, Schellhorn B, Pieren M, Lociuro S, Gitzinger M, Tigges M, Bumann D, Kemmer C. 2019. Dissecting colistin resistance mechanisms in extensively drug-resistant Acinetobacter baumannii clinical isolates. MBio 10:e01083-19. doi:10.1128/mBio.01083-19 31311879 PMC6635527

[10] Yahav D, Giske CG, Grāmatniece A, Abodakpi H, Tam VH, Leibovici L. 2020. New β-lactam-β-lactamase inhibitor combinations. Clin Microbiol Rev 34:e00115-20. doi:10.1128/CMR.00115-20 PMC7667665 33177185

[11] IDSA. 2023. Guidance on the treatment of antimicrobial resistant gram-negative infections. Available from: https://www.idsociety.org/practice-guideline/amr-guidance. Retrieved 5 Jan 2024.

[12] Pachón-Ibáñez ME, Docobo-Pérez F, López-Rojas R, Domínguez-Herrera J, Jiménez-Mejias ME, García-Curiel A, Pichardo C, Jiménez L, Pachón J. 2010. Efficacy of rifampin and its combinations with imipenem, sulbactam, and colistin in experimental models of infection caused by imipenem-resistant Acinetobacter baumannii. Antimicrob Agents Chemother 54:1165–1172. doi:10.1128/AAC.00367-09 20047914 PMC2825983

[13] Song JY, Cheong HJ, Lee J, Sung AK, Kim WJ. 2009. Efficacy of monotherapy and combined antibiotic therapy for carbapenem-resistant Acinetobacter baumannii pneumonia in an immunosuppressed mouse model. Int J Antimicrob Agents 33:33–39. doi:10.1016/j.ijantimicag.2008.07.008 18835761

[14] Wolff M, Joly-Guillou M-L, Farinotti R, Carbon C. 1999. In vivo efficacies of combinations of beta-lactams, beta-lactamase inhibitors, and rifampin against Acinetobacter baumannii in a mouse pneumonia model. Antimicrob Agents Chemother 43:1406–1411. doi:10.1128/AAC.43.6.1406 10348761 PMC89287

[15] Ju YG, Lee HJ, Yim HS, Lee M-G, Sohn JW, Yoon YK. 2022. In vitro synergistic antimicrobial activity of a combination of meropenem, colistin, tigecycline, rifampin, and ceftolozane/tazobactam against carbapenem-resistant Acinetobacter baumannii. Sci Rep 12:7541. doi:10.1038/s41598-022-11464-6 35534512 PMC9085847

[16] Bassetti M, Repetto E, Righi E, Boni S, Diverio M, Molinari MP, Mussap M, Artioli S, Ansaldi F, Durando P, Orengo G, Bobbio Pallavicini F, Viscoli C. 2007. Colistin and rifampicin in the treatment of multidrug-resistant Acinetobacter baumannii infections. J Antimicrob Chemother 61:417–420. doi:10.1093/jac/dkm509 18174197

[17] Saballs M, Pujol M, Tubau F, Peña C, Montero A, Domínguez MA, Gudiol F, Ariza J. 2006. Rifampicin/imipenem combination in the treatment of carbapenem-resistant Acinetobacter baumannii infections. J Antimicrob Chemother 58:697–700. doi:10.1093/jac/dkl274 16895941

[18] Durante-Mangoni E, Signoriello G, Andini R, Mattei A, De Cristoforo M, Murino P, Bassetti M, Malacarne P, Petrosillo N, Galdieri N, Mocavero P, Corcione A, Viscoli C, Zarrilli R, Gallo C, Utili R. 2013. Colistin and rifampicin compared with colistin alone for the treatment of serious infections due to extensively drug-resistant Acinetobacter baumannii: a multicenter, randomized clinical trial. Clin Infect Dis 57:349–358. doi:10.1093/cid/cit253 23616495

[19] Karakonstantis S, Ioannou P, Samonis G, Kofteridis DP. 2021. Systematic review of antimicrobial combination options for pandrug-resistant Acinetobacter baumannii. Antibiotics (Basel) 10:1344. doi:10.3390/antibiotics10111344 34827282 PMC8615225

[20] Lee B, Yan J, Ulhaq A, Miller S, Seo W, Lu P, She R, Spellberg B, Luna B. 2021. In vitro activity of rifabutin and rifampin against antibiotic-resistant Acinetobacter baumannii, Escherichia coli, Staphylococcus aureus, Pseudomonas aeruginosa, and Klebsiella pneumoniae*.* mSphere 6:e0092021. doi:10.1128/msphere.00920-21 34817233 PMC8612264

[21] Karballaei-Mirzahosseini H, Kaveh-Ahangaran R, Shahrami B, Rouini MR, Najafi A, Ahmadi A, Sadrai S, Mojtahedzadeh A, Najmeddin F, Mojtahedzadeh M. 2022. Pharmacokinetic study of high-dose oral rifampicin in critically Ill patients with multidrug-resistant Acinetobacter baumannii infection. DARU J Pharm Sci 30:311–322. doi:10.1007/s40199-022-00449-5 PMC9715901 36069988

[22] Lepe JA, García-Cabrera E, Gil-Navarro MV, Aznar J. 2012. Rifampin breakpoint for Acinetobacter baumannii based on pharmacokinetic-pharmacodynamic models with Monte Carlo simulation. Rev Esp Quimioter 25:134–138. 22707102

[23] Ruslami R, Nijland HMJ, Alisjahbana B, Parwati I, van Crevel R, Aarnoutse RE. 2007. Pharmacokinetics and tolerability of a higher rifampin dose versus the standard dose in pulmonary tuberculosis patients. Antimicrob Agents Chemother 51:2546–2551. doi:10.1128/AAC.01550-06 17452486 PMC1913243

[24] Garcia-Prats AJ, Svensson EM, Winckler J, Draper HR, Fairlie L, van der Laan LE, Masenya M, Schaaf HS, Wiesner L, Norman J, Aarnoutse RE, Karlsson MO, Denti P, Hesseling AC. 2021. Pharmacokinetics and safety of high-dose rifampicin in children with TB: the opti-rif trial. J Antimicrob Chemother 76:3237–3246. doi:10.1093/jac/dkab336 34529779 PMC8598292

[25] Seijger C, Hoefsloot W, Bergsma-de Guchteneire I, Te Brake L, van Ingen J, Kuipers S, van Crevel R, Aarnoutse R, Boeree M, Magis-Escurra C. 2019. High-dose rifampicin in tuberculosis: experiences from a Dutch tuberculosis centre. PLoS One 14:e0213718. doi:10.1371/journal.pone.0213718 30870476 PMC6417786

[26] Byrne JM, Waack U, Weinstein EA, Joshi A, Shurland SM, Iarikov D, Bulitta JB, Diep BA, Guina T, Hope WW, Lawrenz MB, Lepak AJ, Luna BM, Miesel L, Phipps AJ, Walsh TJ, Weiss W, Amini T, Farley JJ. 2020. FDA public workshop summary: advancing animal models for antibacterial drug development. Antimicrob Agents Chemother 65:e01983-20. doi:10.1128/AAC.01983-20 33106262 PMC7927847

[27] Farrington N, Dubey V, Johnson A, Horner I, Stevenson A, Unsworth J, Jimenez-Valverde A, Schwartz J, Das S, Hope W, Darlow CA. 2024. Molecular pharmacodynamics of meropenem for nosocomial pneumonia caused by Pseudomonas aeruginosa. MBio 15:e0316523. doi:10.1128/mbio.03165-23 38236031 PMC10865990

[28] Etienne M, Croisier D, Charles P-E, Lequeu C, Piroth L, Portier H, Drlica K, Chavanet P. 2004. Effect of low-level resistance on subsequent enrichment of fluoroquinolone-resistant Streptococcus pneumoniae in rabbits. J Infect Dis 190:1472–1475. doi:10.1086/423853 15378440

[29] EUCAST Breakpoint Tables 12. 2024 Available from: https://www.eucast.org/clinical_breakpoints

[30] Jaruratanasirikul S, Sriwiriyajan S, Punyo J. 2005. Comparison of the pharmacodynamics of meropenem in patients with ventilator-associated pneumonia following administration by 3-hour infusion or bolus injection. Antimicrob Agents Chemother 49:1337–1339. doi:10.1128/AAC.49.4.1337-1339.2005 15793108 PMC1068632

[31] Aarnoutse RE, Kibiki GS, Reither K, Semvua HH, Haraka F, Mtabho CM, Mpagama SG, van den Boogaard J, Sumari-de Boer IM, Magis-Escurra C, Wattenberg M, Logger JGM, Te Brake LHM, Hoelscher M, Gillespie SH, Colbers A, Phillips PPJ, Plemper van Balen G, Boeree MJ, PanACEA Consortium. 2017. Pharmacokinetics, tolerability, and bacteriological response of rifampin administered at 600, 900, and 1,200 milligrams daily in patients with pulmonary tuberculosis. Antimicrob Agents Chemother 61:e01054-17. doi:10.1128/AAC.01054-17 28827417 PMC5655063

[32] Jayaram R, Gaonkar S, Kaur P, Suresh BL, Mahesh BN, Jayashree R, Nandi V, Bharat S, Shandil RK, Kantharaj E, Balasubramanian V. 2003. Pharmacokinetics-pharmacodynamics of rifampin in an aerosol infection model of tuberculosis. Antimicrob Agents Chemother 47:2118–2124. doi:10.1128/AAC.47.7.2118-2124.2003 12821456 PMC161844

[33] Rangel K, De-Simone SG. 2024. Treatment and management of Acinetobacter pneumonia: lessons learned from recent world event. Infect Drug Resist 17:507–529. doi:10.2147/IDR.S431525 38348231 PMC10860873

[34] Rando E, Cutuli SL, Sangiorgi F, Tanzarella ES, Giovannenze F, De Angelis G, Murri R, Antonelli M, Fantoni M, De Pascale G. 2023. Cefiderocol-containing regimens for the treatment of carbapenem-resistant A. baumannii ventilator-associated pneumonia: a propensity-weighted cohort study. JAC Antimicrob Resist 5:dlad085. doi:10.1093/jacamr/dlad085 37484029 PMC10359102

[35] Chastre J, Fagon JY, Bornet-Lecso M, Calvat S, Dombret MC, al Khani R, Basset F, Gibert C. 1995. Evaluation of bronchoscopic techniques for the diagnosis of nosocomial pneumonia. Am J Respir Crit Care Med 152:231–240. doi:10.1164/ajrccm.152.1.7599829 7599829

[36] Andes D, Craig WA. 2002. Pharmacodynamics of the new fluoroquinolone gatifloxacin in murine thigh and lung infection models. Antimicrob Agents Chemother 46:1665–1670. doi:10.1128/AAC.46.6.1665-1670.2002 12019073 PMC127205

[37] Isberg B, Paul C, Jönsson L, Svahn U. 1991. Myocardial toxicity of high-dose cyclophosphamide in rabbits treated with daunorubicin. Cancer Chemother Pharmacol 28:171–180. doi:10.1007/BF00685505 1649704

[38] Jain S, Srivastava D, Pandey G, Shrivastav A. 2006. Cytotoxic effect of cyclophosphamide in the kidney and intestine of the rabbit. Ind Vet J 83:730–731. doi:10.1111/j.1751-0813.2005.tb11577.x

[39] Ray S, Chowdhury P, Pandit B, Ray SD, Das S. 2010. Exploring the antiperoxidative potential of morin on cyclophosphamide and flutamide-induced lipid peroxidation and changes in cholesterol profile in rabbit model. Acta Pol Pharm 67:35–44. 20210077

[40] Croisier D. 2023. AMR Conference 2023. oral session. Basel.

[41] Imberti R, Cusato M, Villani P, Carnevale L, Iotti GA, Langer M, Regazzi M. 2010. Steady-state pharmacokinetics and BAL concentration of colistin in critically Ill patients after IV colistin methanesulfonate administration. Chest 138:1333–1339. doi:10.1378/chest.10-0463 20558557

[42] Zhao X, Drlica K. 2002. Restricting the selection of antibiotic-resistant mutant bacteria: measurement and potential use of the mutant selection window. J Infect Dis 185:561–565. doi:10.1086/338571 11865411

[43] Nordqvist H, Nilsson LE, Claesson C. 2016. Mutant prevention concentration of colistin alone and in combination with rifampicin for multidrug-resistant Acinetobacter baumannii. Eur J Clin Microbiol Infect Dis 35:1845–1850. doi:10.1007/s10096-016-2736-3 27510182 PMC5059421

[44] Pesta M, Datler P, Scheriau G, Wohlrab P, Eberl S, Lackner E, Franz C, Jäger W, Maier-Salamon A, Zeitlinger M, Tschernko E. 2023. Comparison of antibiotic protein binding in human plasma vs. rabbit plasma. bioRxiv. doi:10.1101/2023.03.23.534048

[45] Rifat D, Prideaux B, Savic RM, Urbanowski ME, Parsons TL, Luna B, Marzinke MA, Ordonez AA, DeMarco VP, Jain SK, Dartois V, Bishai WR, Dooley KE. 2018. Pharmacokinetics of rifapentine and rifampin in a rabbit model of tuberculosis and correlation with clinical trial data. Sci Transl Med 10:eaai7786. doi:10.1126/scitranslmed.aai7786 29618565 PMC5969904

[46] Croisier D, Chavanet P, Lequeu C, Ahanou A, Nierlich A, Neuwirth C, Piroth L, Duong M, Buisson M, Portier H. 2002. Efficacy and pharmacodynamics of simulated human-like treatment with levofloxacin on experimental pneumonia induced with penicillin-resistant pneumococci with various susceptibilities to fluoroquinolones. J Antimicrob Chemother 50:349–360. doi:10.1093/jac/dkf131 12205059

